# Prognostic Significance of Myocardial Ischemia Detected by Single-Photon Emission Computed Tomography in Children with Hypertrophic Cardiomyopathy

**DOI:** 10.1007/s00246-021-02570-9

**Published:** 2021-03-09

**Authors:** Lidia Ziolkowska, Agnieszka Boruc, Dorota Sobielarska-Lysiak, Agnieszka Grzyb, Joanna Petryka-Mazurkiewicz, Łukasz Mazurkiewicz, Grazyna Brzezinska-Rajszys

**Affiliations:** 1 Department of Cardiology, The Children’s Memorial Health Institute, Al. Dzieci Polskich 20, 04-730 Warszawa, Poland; 2 Department of Nuclear Medicine, The Children’s Memorial Health Institute, Al. Dzieci Polskich 20, 04-730 Warszawa, Poland; 3 Magnetic Resonance Unit, Department of Cardiomyopathies, National Institute of Cardiology, 04-628 Warsaw, Poland; 4 Department of Coronary and Structural Heart Diseases, National Institute of Cardiology, 04-628 Warsaw, Poland

**Keywords:** Children, Hypertrophic cardiomyopathy, Myocardial ischemia, Prognosis

## Abstract

Myocardial ischemia caused by microvascular dysfunction is an important pathophysiologic component of hypertrophic cardiomyopathy (HCM), promoting myocardial fibrosis, adverse left ventricular remodeling, and impacting on clinical course and outcome in HCM patients. The aim of study was to assess the prevalence and clinical significance of myocardial ischemia in children with HCM using 99mTc-MIBI single-photon emission computed tomography (SPECT). Ninety-one children with HCM, median age 13.6 years, underwent SPECT evaluation from 2006 to 2017. Imaging was performed at rest and after maximal exercise. Myocardial perfusion defects were identified in 70 children (76.9%; group I), median age 13.8 years. Fixed perfusion defects were evident in 22 of them, while reversible at rest in 48. In 21 children (23.1%; group II), median age 11 years, myocardial perfusion defects were not detected. Patient demographics, echocardiography, resting electrocardiogram (ECG), 24-h Holter ECG, myocardial fibrosis in cardiovascular magnetic resonance imaging, and cardiovascular events were analyzed and compared between the groups. During follow-up at a median of 8.3 years in children with myocardial ischemia, clinical endpoints occurred more often (47 vs. 5; *p* = 0.02) and more patients reached a clinical endpoint (28 [40%] vs. 3 [14.3%]; *p* = 0.036). In children with myocardial ischemia, myocardial fibrosis was observed with greater frequency. Myocardial perfusion defects may reflect an ischemic process which (1) affects the clinical manifestations and (2) is an important predictor of adverse clinical events and risk of death in children with HCM. Myocardial ischemia in HCM patients frequently correlates with myocardial fibrosis.

## Introduction

Hypertrophic cardiomyopathy (HCM) is the second most common cardiomyopathy in children, with an estimated annual incidence of 0.24–0.47 per 100,000 [1, 2]. The etiology of disease is more heterogeneous than seen in adult populations, with as many as 30% of patients having an inborn error of metabolism, malformation syndrome, or neuromuscular disease [3, 4]. The long-term outcome in children with HCM is highly variable and has been shown to depend partly on the age of presentation and underlying etiology [5–7]. The risk factors for sudden death and for heart failure-related death are different in childhood HCM [7]. Outside of infancy, the most frequent cause of mortality is sudden cardiac death (SCD). The morphological features of HCM are left ventricular (LV) diastolic dysfunction due to hypertrophy and stiffening of the myocardium [8], decreased myocardial perfusion (MP), and fibrosis [9]. Some myocardial perfusion single-photon emission computed tomography (SPECT) studies have suggested that the presence of regional perfusion defects could have prognostic implications in adult patients with HCM [10, 11]. The use of nuclear medicine techniques utilizing 99mTc-MIBI SPECT in the diagnosis of HCM in children seems to be helpful in determining the prognosis and assessing the risk of an unfavorable course of the disease [12]. The prognostic value of late gadolinium enhancement (LGE) assessed fibrosis in HCM remains the subject of research [13], and myocardial ischemia has been proposed as an earlier marker of disease and influencing the prognosis [14]. The aim of this study was to assess the prevalence and clinical significance of myocardial ischemia, underlining its impact on adverse prognosis in children with HCM.

## Methods

### Study Population

From January 2006 to November 2017 we prospectively enrolled pediatric patients with diagnosed HCM hospitalized in the cardiology department. All our HCM patients underwent 99mTc-MIBI SPECT evaluation and then were followed prospectively for a median of 8.3 years to assess the occurrence of cardiovascular events. Criteria for inclusion in the study were age < 18 years at time of diagnosis and echocardiographic evidence of LV hypertrophy defined as a maximal left ventricular wall thickness greater than two standard deviations above the body surface area-corrected mean (z-score ≥ 2) and not explained by abnormal loading conditions [15, 16]. The Institutional Ethics Committee approved this study. Informed consent was obtained from all individual participants included in the study.

### Data Collection

Patients demographics, clinical symptoms, as well as the results of echocardiography, resting and ambulatory 12 lead electrocardiograms (ECG), CMR, and 99mTc-MIBI SPECT at rest and after exercise test were collected. Cardiac examinations were repeatedly performed during the follow-up period.

In all children, routinely planned cardiac examinations (echocardiography, resting and ambulatory 12 lead ECG) and clinical evaluation every 6 months were performed. In the case of impairment of the general condition, onset of clinical symptoms and disease progression, cardiac tests, including repeated CMR, were performed more frequently, according to individual indications.

For analysis, the most recent or latest results before the endpoint were chosen. The period from diagnosis of HCM to the most recent study was median 10.8 (6.5–13.2) years. The median period between SPECT and recent results was 8.3 (4.3–10.8) years.

Sudden cardiac death, unexplained syncope and non-sustained ventricular tachycardia (NSVT) were defined as recommended in the literature [1]. Echocardiographic measurements were made according to current guidelines [15]. Left ventricular outflow tract (LVOT) obstruction was defined as a peak instantaneous gradient ≥ 30 mmHg at rest [15]. Enlargement of left atrium (LA) was defined as z-score ≥ 2 [2]. Maximal left ventricular wall thickness and LA diameter measurements are expressed in millimeters and as body surface area-corrected z-scores [16, 17].

Cardiovascular magnetic resonance imaging (CMR) was performed using a 1.5-T scanner (Sonata and Avanto fit, Siemens, Germany). Cine images were acquired by the breath-hold, electrocardiographic-gated, segmented k-space steady-state free-precession technique using 25 phases per cardiac cycle. LGE images were obtained in the long-axis and short-axis imaging planes using a breath-hold-segmented inversion recovery sequence implemented 10–15 min after intravenous administration of 0.1 mmol/kg of gadobutrol (Gadovist, Bayer, Berlin, Germany); gadodiamide (Omniscan, GE Healthcare, United Kingdom) was used instead gadobutrol if the patient was under 2 years of age. The presence of left ventricular LGE and myocardial fibrosis was determined using visual assessment by two independent observers and if positive, quantified. Quantification was performed both as a signal intensity threshold of > 6 SD above remote myocardium. The extent of LGE is presented in grams and as the percentage of total LV mass [18].

The 99mTc-MIBI SPECT was performed for evaluation of MP and prevalence of perfusion defects indicative of myocardial ischemia. The tracer used was sestamibi (MIBI) labeled with technetium 99 m. The 99mTc‑MIBI chelate was administered intravenously at a dose of 111 to 740 MBq per kilogram of body weight [19]. MP scans were performed at rest and after maximal exercise test using Siemens Dual Head Nuclear Gamma Camera, CEDAR cardiac software, ECG Gate (G-SPECT), with automatic reconstruction of sections and creation of polar maps—bull’s eye. A two-day protocol was used, scanning time each day 15 min. In order to minimize radiation exposure and increase radiation safety in the studied group of children, we used a two-day examination protocol (SPECT at rest on Monday and SPECT at exercise on Wednesday). The abovementioned doses of 99mTc-MIBI chelate were the maximum doses, we used doses strictly defined in relation to the child's body weight, it is 0.3 milicurie (mCi) i.e., 11.1 MBq per kilogram of body weight for the first and second part of the test (SPECT at rest and during exercise). The maximum dose of 740 MBq (20 mCi) corresponds to the radiation dose equivalent to absorbed 12.5 milliSievert (mSv). The chemical half-life (T 1/2) of the radiotracer is 6 h, while the biological T 1/2 of the radiotracer is shorter, as it is excreted in the bile through the gastrointestinal tract and in the urine. The acquisition and reconstruction technique of G-SPECT myocardial perfusion was exactly described in the guidelines [20]. Stress SPECT was evaluated after physiological exercise on a treadmill according to a modified Bruce protocol. Maximum intensity of exercise was limited by patient fatigue or clinical symptoms. Patients fasted at least eight hours prior to the scheduled test. Medications that can affect the course of the exercise (beta blockers, calcium channel blockers) had been discontinued 48 h prior to examination. Normal radiotracer distribution in the myocardium pointed to the lack of perfusion disorders and absence of myocardial ischemia. A reversible perfusion defect was defined as an area of decreased radiotracer accumulation during the exercise study which resolves itself entirely while at rest. Fixed perfusion defects were found when myocardial perfusion defects were present in both exercise and rest [21].

### Events Data

Patient events were recorded by communication with children‘s parents. Medical records were reviewed after attendance in outpatient clinics or hospital stay. No patient was lost to follow-up. Clinical endpoints included these cardiovascular events: SCD, HF-related death, resuscitated cardiac arrest (rCA), appropriate ICD discharges (ICDdx), heart transplant (HTx), NSVT, syncope, and progression of heart failure from New York Heart Association (NYHA) class I or II to class III. Moreover, arrhythmic endpoints was predefined and included: SCD, rCA, adequate ICDdx, NSVT.

### Methods of Analysis

All children were divided into group I (gI) with and group II without myocardial ischemia in SPECT (gII). The presence and extent of the perfusion defects were analyzed by two independent experts and relative myocardial perfusion was assessed. In a descriptive way, the presence of perfusion defects was determined in the following projections: short axis (septal, lateral, anterior, inferior wall), vertical long axis (base, apex, anterior, inferior wall) and horizontal long axis (base, apex, septal, lateral wall). The presence of perfusion defects indicated a significantly lower concentration of 99mTc-MIBI in the myocardium. In the case of fixed myocardial perfusion defects SPECT images showed significantly lower concentration of 99mTc‑MIBI in stress and in rest images indicating severe myocardial ischemia. In the case of reversible perfusion defects, SPECT scans showed significant radiotracer accumulation improvement in rest images.

The demographic, clinical, echocardiographic, CMR parameters, and occurrence of endpoints were analyzed and compared between the two groups.

### Statistics

Statistical analysis was performed using Statistica software. Continuous variables are described as median with interquartile range (25th to 75th centile). Nominal variables are presented as a numerical value and the percentage of the study group. Continuous variables were compared by *U* Mann–Whitney test. Binary variables were compared using Fisher test or chi-square test. The Kaplan–Meier survival curves were constructed to compare the groups with and without myocardial ischemia and with fixed or reversible perfusion defects in terms of reaching endpoints. Values of *p* < 0.05 were considered statistically significant.

## Results

### Baseline Characteristics

**Fig. 1:**
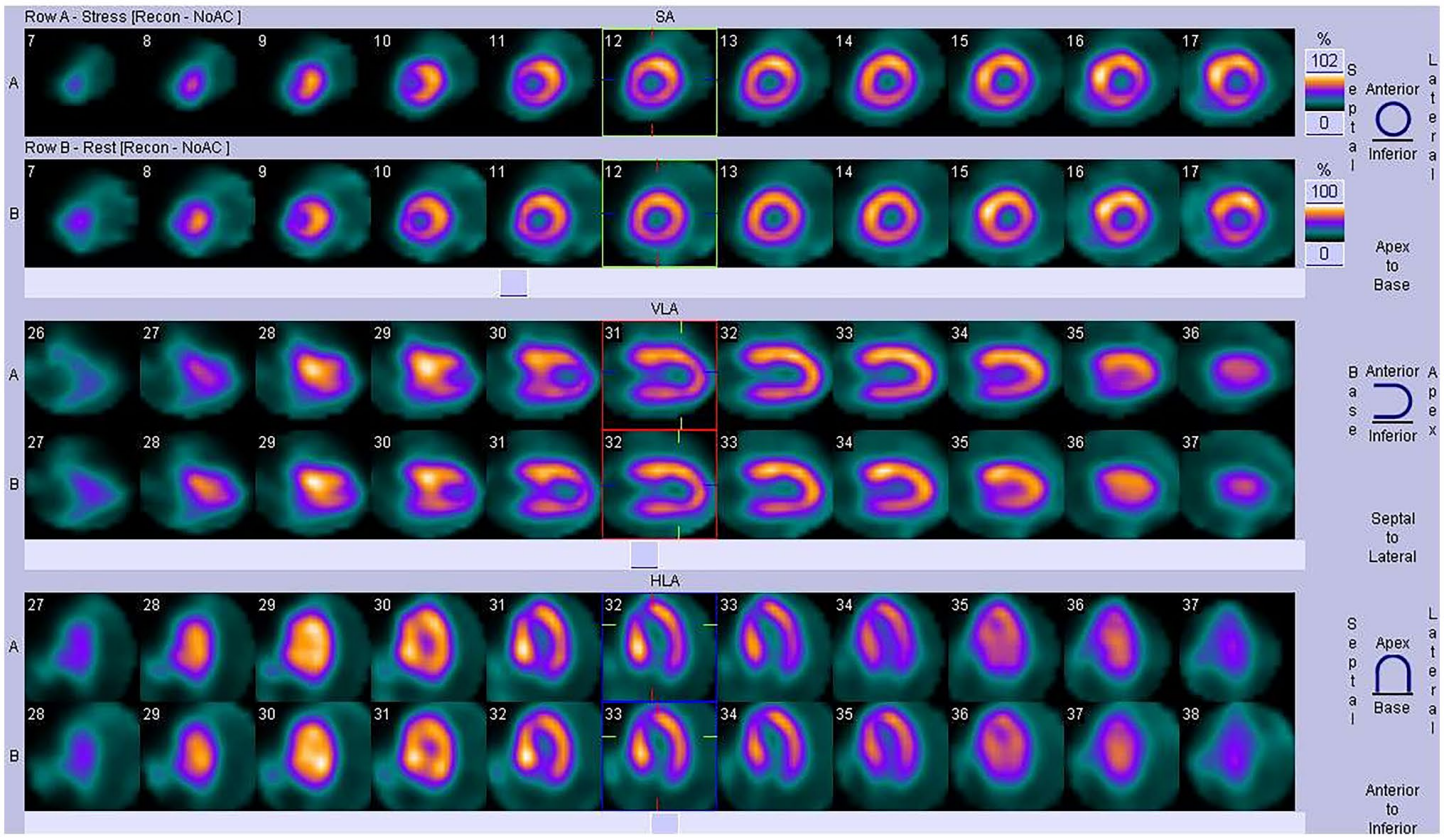
SPECT imaging: patient with septal and left ventricular posterior wall hypertrophy. Fixed perfusion defects were detected during exercise in the basal segments of septum and in the left ventricular inferior wall. Perfusion defects not resolved at rest

**Fig. 2:**
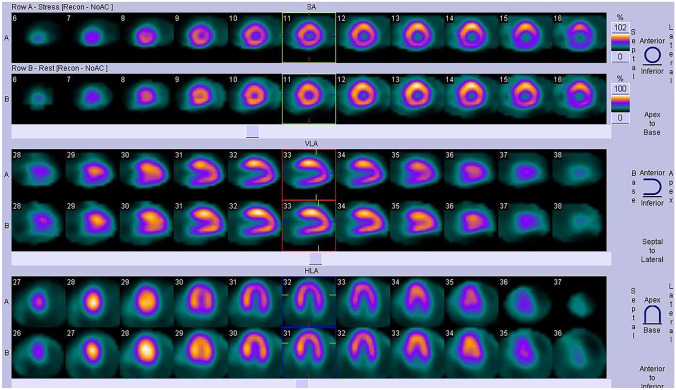
SPECT imaging: patient with asymmetric septal hypertrophy. Perfusion defects were detected only during exercise in the basal segments of septum and in the left ventricular inferolateral wall. Perfusion defects completely resolved at rest

A total of 91 patients with HCM, median age 13.6 (10.3–15.8) years, 64% male, in NYHA class I or II, underwent SPECT evaluation from January 2006 to November 2017. Patients were followed prospectively for 8.3 (4.3–10.8) years. MP defects were identified in 70 of the 91 children (76.9%; gI), median age 13.8 years (10.7–15.9). Fixed perfusion defects were evident in 22 (31.4%) patients (Fig. 1). In 48 (68.5%) children, perfusion defects were present only during exercise and they completely resolved at rest (Fig. 2). In 21 children (23.1%; gII), median age 11 years (8.8–15.1), myocardial perfusion defects were not detected. In the group of children with perfusion defects in the SPECT study (70 pts), 42 patients (60%) had ST depression in the ECG record during the exercise test, while in the remaining 28 children (40%), no such changes were found. Among 21 children without perfusion defects in SPECT, changes in exercise ECG in the form of ST depression were found in five patients (24%), while there were no ST segment depression in the remaining 16 (76%) children. As many as 58% of patients had myocardial enhancement on late gadolinium imaging. In patients with LGE, the median extent was 2.0% (IQR, 1.3–5.7%) of LV mass.

**Fig. 3:**
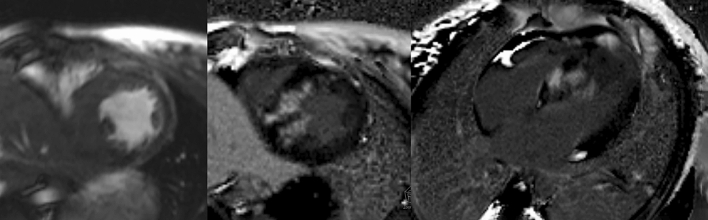
Late gadolinium enhancement (LGE) on cardiovascular magnetic resonance (CMR) imaging in a child with hypertrophic cardiomyopathy. LEFT: cine image shows asymmetrical hypertrophy; MIDDLE: short-axis LGE image shows extensive mid-wall fibrosis; RIGHT: long-axis four-chamber LGE image confirms extensive mid-wall LGE in the septum

On echocardiography, the median maximal LV wall thickness z-score was 8.7 (gI), while the gII median was 6.6 (*p* = 0.007). A comparable number of children in both groups experienced LVOTO. The median LA diameter z-score did not differ significantly between the two groups studied (3.4 [gI] and 2.6 [gII]). Fibrosis in CMR was found at a significantly greater frequency in children with perfusion defects compared to children without myocardial ischemia (66% vs. 25%; *p* = 0.02), (Fig. 3). Clinical characteristics of the patient population are presented in Table 1.

**Table 1:** Clinical characteristics of the patient population

	Analyzed parameters	All patients (n = 91)	Patients with myocardial ischemia (n = 70, 76.9%)	Patients without myocardial ischemia (n = 21, 23.1%)	p value
Demographic data	Age at diagnosis of HCM (years**)**	10.9 (3.9–14.2)	11.6 (3.7–14.1)	10.2 (6.5–14.2)	0.89
	Age at time of SPECT test (years**)**	13.6 (10.3–15.8)	13.8 (10.7–15.9)	11.0 (8.8–15.1)	0.16
	Follow-up time from HCM diagnosis (years**)**	10.8 (6.5–13.2)	11.0 (7.5–13.6)	9.4 (4.3–12.0)	0.09
	Follow-up time since SPECT test (years**)**	8.3 (4.3–10.8)	8.2 (5.3–10.7)	8.9 (3.7–10.8)	0.71
Exercise data	Maximum effort (minutes)	6.74 (3.0–13.3)	6.72 (3.0–13.3)	6.79 (5.3–13.1)	0.73
	Maximum activity (METs)	8.2 (4.0–17.1)	8.0 (4.0–17.1)	8.3 (7.0–17.1)	0.68
End point analysis n (%)	Patients with end points	31 (34.1)	28 (40.0)	3 (14.3)	**0.036**
	Number of end points	52	47	5	**0.02**
	SCD	3 (3.3)	3 (4.3)	0	NS
	ICD dx	6 (6.6)	6 (8.6)	0	0.33
	rCA	1 (1.1)	1 (1.4)	0	NS
	HF-related death	1 (1.1)	1 (1.4)	0	NS
	HTx	3 (3.3)	2 (2.9)	1 (4.8)	0.55
	Syncope	12 (13.2)	10 (14.3)	2 (9.5)	0.73
	nsVT	15 (16.5)	15 (21.4)	0	**0.018**
	Progression from NYHA class I or II to class III	11 (12.1)	9 (12.9)	2 (9.5)	NS
Other data	Fibrosis in CMR	36/62 (58.1%)	33/50 (66.0%)	3/12 (25.0%)	**0.02**
	LVOTO ≥ 30 mmHg	19 (20.9%)	15 (21.4%)	4 (19.0%)	NS
	Maximal wall thickness z-score	7.8 (4.7–13.3)	8.7 (5.2–15.4)	6.4 (3.1–8.4)	**0.007**
	LA size (LAd**)** z-score	3.4 (1.3–6.5)	3.4 (1.4–6.5)	2.6 (0.4–5.4)	0.39

Data expressed as median (IQR) or frequencies (percentages). *NS* non-significant

*METs* metabolic equivalents; *LAd* left atrial diameter, *SCD* sudden cardiac death, *nsVT* non-sustained ventricular tachycardia, *LVOTO* left ventricular outflow tract obstruction, *HTx* heart transplantation, *HF* heart failure, *rCA* resuscitated cardiac arrest, *ICDdx* appropriate ICD discharges

### Outcomes

During follow-up, clinical endpoints occurred significantly more often in children with recognized perfusion defects and myocardial ischemia (47 vs. 5; *p* = 0.02) and significantly more patients reached a clinical endpoint (28 [40%] vs. 3 [14.3%]; *p* = 0.036).

**Fig. 4:**
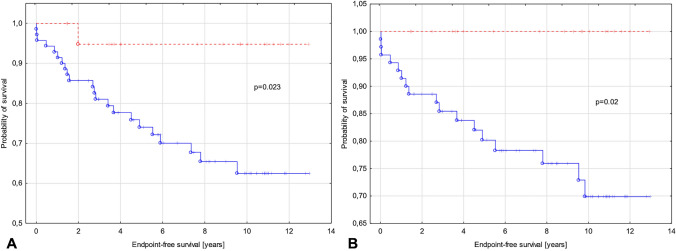
Kaplan-Meyer curves for groups with (blue) and without myocardial ischemia (red). **a** Endpoints include sudden cardiac death, resuscitated sudden cardiac arrest, adequate ICD discharge, heart failure-related death, heart transplant, syncope, and non-sustained ventricular tachycardia. **b** Endpoints include arrhythmic events such as sudden cardiac death, resuscitated sudden cardiac arrest, adequate ICD discharge, and non-sustained ventricular tachycardia

**Fig. 5:**
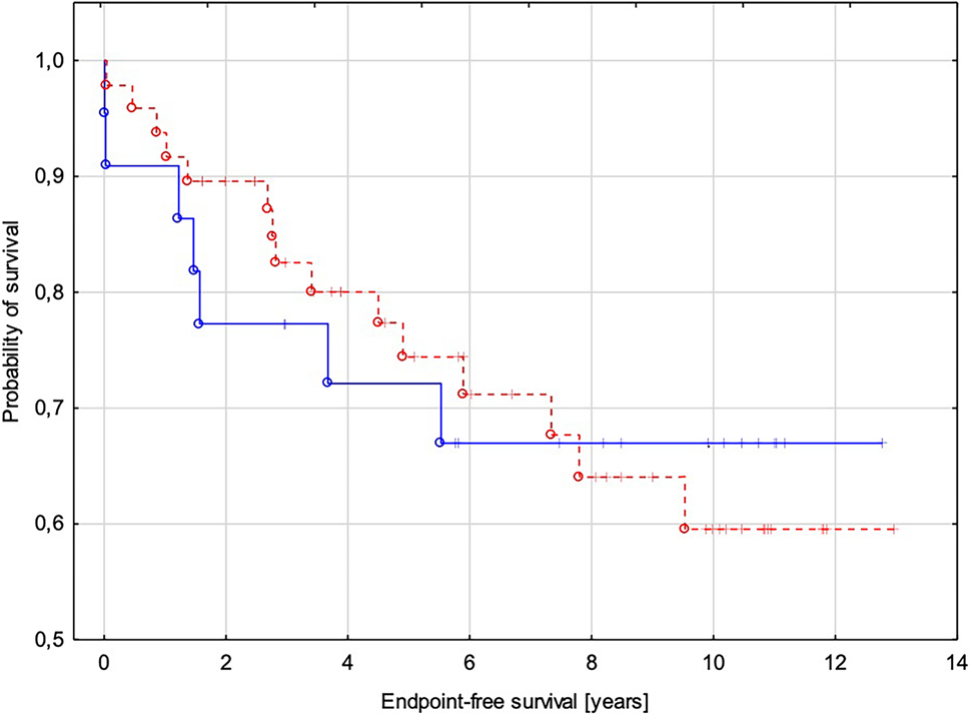
Kaplan-Meyer curves for groups with fixed (blue) and reversible perfusion defect (red) showing no substantial differences between groups. Endpoints include sudden cardiac death, resuscitated sudden cardiac arrest, adequate ICD discharge, heart failure-related death, heart transplant, syncope, and non-sustained ventricular tachycardia

In children with perfusion defects, the following cardiovascular events occurred: SCD (*n* = 3), rCA (*n* = 1), ICDdx (*n* = 6), NSVT (*n* = 15), syncope (*n* = 10), progression of heart failure from NYHA class I or II to class III (*n* = 9), HF-related death (*n* = 1), and HTx (*n* = 2). In children with myocardial ischemia, significantly more frequent myocardial fibrosis was observed (66% vs. 25%; *p* = 0.02). The data are presented in Table 1. The survival analyses including all endpoints or only arrhythmic endpoints (Fig. 4a, b) showed statistically significant differences between groups with diagnosed myocardial ischemia and without it. Kaplan-Meyer curves for groups with fixed and reversible perfusion defect showing no substantial differences between groups (Fig. 5).

## Discussion

Myocardial ischemia can occur in patients with HCM in the absence of coronary artery disease [22] and is associated with abnormal MP [23]. Myocardial ischemia caused by microvascular dysfunction is an important pathophysiologic component of HCM, promoting myocardial fibrosis, adverse LV remodeling, and impacting clinical course and outcome in HCM patients [10, 24]. Autopsy studies have shown structural abnormalities of the intramural coronary arterioles characterized by intimal thickening and dense perivascular collagen with decreased luminal cross-sectional area, which is thought to represent the substrate for microvascular dysfunction [25, 26]. Blunted myocardial blood flow during stress is the functional consequence of microvascular dysfunction and can be detected as regions of hypoperfusion with various imaging techniques [27]. The mechanisms and relation of the development of hypoperfused areas and fibrosis are, however, not fully understood [28–30]. It is thought that small-vessel coronary artery disease may be an important driver of replacement fibrosis by repetitive bouts of microvascular ischemia contributing to myocyte cell death [31].

The LGE CMR sequences identify the persistence of gadolinium-based contrast agents within areas of fibrosis due to their accumulation and delayed washout from an expanded extracellular space [32]. A growing body of research addresses the use of CMR-derived LGE for HCM risk stratification [33, 34]. The failure of myocardial blood flow to increase adequately on demand in patients with HCM is clinically relevant in that it predisposes them to myocardial ischemia, which has been implicated in the pathogenesis of syncope, an abnormal blood pressure response to exercise, LV systolic dysfunction, and sudden death [35]. Prognostic MP studies in patients with HCM are scarce, especially in the pediatric population. Von Dohlen [36] observed that thallium perfusion abnormalities were strongly associated with potentially lethal arrhythmias while Dilsizian [35] reported that reversible thallium defects were significantly more common in patients with a history of cardiac arrest. Yamada [37] found that fixed defects were significantly more common in patients with syncope. The findings of additional authors show that severe perfusion abnormalities are independently predictive of death [35] and development of heart failure [38].

In agreement with other investigations [16, 39, 40], our study showed that most patients had various degrees of impairment in myocardial blood flow and MP defects in SPECT.

During follow-up lasting longer than eight years, 40% of patients with myocardial ischemia had cardiovascular events. Moreover, an association was found between the presence of MP abnormalities and the prevalence of cardiovascular endpoints. It should also be emphasized that the number of endpoints was significantly higher in children with MP defects, which confirms current literature reporting that, in patients with HCM, the degree of microvascular dysfunction is a strong, independent predictor of clinical deterioration and death [28].

The results of our study showed that myocardial ischemia was strongly associated with an unfavorable outcome, which was confirmed by the analysis of the survival curves. This finding is consistent with the results of similar studies by others [34, 41]. It is noteworthy that Kaplan-Meyer curves for groups with fixed and reversible perfusion defect showing no substantial differences between them, which requires further research in a larger group of patients. It is thought that MP disorders and subsequent fibrosis may act as arrhythmogenic substrates as there is a proven association between LGE and ventricular arrhythmias. In our study, the subgroup of patients with perfusion abnormalities experienced a significantly greater number of episodes of NSVT in comparison to children with normal MP, a finding also consistent with reports of other authors [42]. It was noteworthy that all three sudden deaths and one from heart failure were in only the myocardial ischemia group. Similarly, arrhythmic events such as rCA and adequate ICD discharge occurred only in children with MP defects. Among the 12 children who experienced syncope, as many as ten were in the group of patients with impaired MP. Progression of heart failure and escalation of NYHA class I and II to III were more frequently observed in children with myocardial ischemia, although heart transplantation was performed in two patients with myocardial ischemia and in one child without it. One of the methods to assess myocardial ischemia in children with hypertrophic cardiomyopathy is the evaluation of exercise-induced ECG changes in the form of ST segment depression during exercise testing. It should be emphasized that in the analyzed group of patients with perfusion defects in the SPECT study, as many as 60% of children had ST- depression provoked by exercise, compared to 24% of patients without perfusion defects in SPECT.

As in the studies of other authors, the maximum LV wall thickness correlated with the presence of perfusion disorders [40]. In children with MP defects, myocardial hypertrophy occurred with significantly greater degree than in patients without them. This finding is of particular relevance since LGE in CMR is observed in 60% to 80% of patients with HCM and is frequently associated with rest perfusion abnormalities [34, 43, 44]. It should be stressed that in our study, as many as 66% of children with MP defects had intramuscular fibrosis expressed by the presence of LGE. As with ours, other studies also proved a close relationship between the severity of microvascular ischemia, the degree of hypertrophy, and the severity of LV fibrosis [20, 45]. Literature reports indicate that myocardial ischemia in children with hypertrophic cardiomyopathy may also be caused by the presence of myocardial bridging. In most cases, they are asymptomatic, therefore, there are only a few studies and individual case reports in the pediatric population. The results of one study showed no evidence that myocardial bridging is an important cause of myocardial perfusion abnormalities [46], while the results of others indicate a relationship between the presence of myocardial bridging, perfusion defects, and the occurrence of adverse cardiovascular events [47]. It remains possible that, in a small group of patients, a severe degree of epicardial coronary compression contributes to myocardial ischemia and can cause chest pain with exercise, syncope, ventricular arrhythmia, and consequently sudden cardiac death. Currently, there is little evidence to suggest that relieving myocardial bridging in children with HCM will improve their symptoms or prognosis. In our center, in accordance with the diagnostic protocol for children with hypertrophic cardiomyopathy, in the case of severe myocardial perfusion defects in SPECT study, the presence of clinical symptoms (chest pain, syncope), ST- segment depression in exercise ECG we perform computed tomography angiography to assess the coronary arteries in terms of the presence of myocardial bridging.

In children with hypertrophic cardiomyopathy and diagnosed myocardial perfusion defects in SPECT, we consider extending the cardiac tests to include computed tomography angiography to assess coronary arteries, modify pharmacological treatment by increasing the dose of beta blocker and recommend low or moderate intensity physical activity. We advise against competitive sports in these children. We determine the indications for ICD implantation on an individual basis.

## Conclusion

In conclusion, we demonstrated that MP defects may reflect an ischemic process which affects clinical manifestations and is an important predictor of adverse clinical events and risk of death in children with HCM. Today's routine diagnostic protocols for HCM do not include testing for myocardial ischemia. It is hoped this and other studies emphasize the value of including this type testing as a key element for developing future risk strategies.
